# Mistakes Matrimonial

**Published:** 1875-06

**Authors:** 


Mistakes Matrimonial.—That the subject is lamentably old we readily admit; but what matters that, so long as humanity is ever new? Marrying and giving in marriage will continue to the end of the world: but, for all that, a great number of marriages prove a mistake. Unfortunately, when men and women have matrimonial designs upon each other, they endeavor to hide their true characters, and, however dissimilar their tastes, to pretend that they are alike. The rules of etiquette are such that a course of deception is rendered surprisingly easy; indeed, so facile, that were there no intention upon the part of those most concerned to dissemble, almost the same end would be attained. Is it not natural to us to display our best attributes to strangers of either sex? Men are often captivated by the seeming amiability of women who are, in reality, shrewish; while women admire the nobility of men who can not define the word "honor." In either case, matrimony follows—afterward misery in five cases out of six. For, after marriage, the true traits of characters begin to show themselves. A scowl, or a sharp word, or a mean action, will not then involve serious consequences. Things that before the knot has been tied would probably bring about separation, after the honeymoon has been passed are quietly received, probably upon the principle that "what can't be cured, must be endured." It must not be understood that we mean to imply that the majority of marriages are entered upon without affection being at the bottom of them. We grant that there is love of a certain kind in many, and very sincere love in some. What we maintain is, that it is brought to fruition under such circumstances that it can not be expected to endure.

